# The prevention of mother-to-child transmission of HIV cascade analysis tool: supporting health managers to improve facility-level service delivery

**DOI:** 10.1186/1756-0500-7-743

**Published:** 2014-10-21

**Authors:** Sarah Gimbel, Joachim Voss, Mary Anne Mercer, Brenda Zierler, Stephen Gloyd, Maria de Joana Coutinho, Florencia Floriano, Maria de Fatima Cuembelo, Jennifer Einberg, Kenneth Sherr

**Affiliations:** Department of Family Child Nursing, University of Washington, Box 357262, Seattle, USA; Department of Global Health, University of Washington, Seattle, USA; Health Alliance International, Seattle, WA USA; Department of Biobehavioral Nursing and Health Systems, University of Washington, Seattle, USA; Health Alliance International, Beira, Mozambique; University of Eduardo Mondlane, Maputo, Mozambique; Consultant, Seattle, USA

**Keywords:** HIV, PMTCT, Mozambique, Cascade analysis, Implementation science, Systems analysis and improvement

## Abstract

**Background:**

The objective of the prevention of Mother-to-Child Transmission (pMTCT) cascade analysis tool is to provide frontline health managers at the facility level with the means to rapidly, independently and quantitatively track patient flows through the pMTCT cascade, and readily identify priority areas for clinic-level improvement interventions. Over a period of six months, five experienced maternal-child health managers and researchers iteratively adapted and tested this systems analysis tool for pMTCT services. They prioritized components of the pMTCT cascade for inclusion, disseminated multiple versions to 27 health managers and piloted it in five facilities. Process mapping techniques were used to chart PMTCT cascade steps in these five facilities, to document antenatal care attendance, HIV testing and counseling, provision of prophylactic anti-retrovirals, safe delivery, safe infant feeding, infant follow-up including HIV testing, and family planning, in order to obtain site-specific knowledge of service delivery.

**Results:**

Seven pMTCT cascade steps were included in the Excel-based final tool. Prevalence calculations were incorporated as sub-headings under relevant steps. Cells not requiring data inputs were locked, wording was simplified and stepwise drop-offs and maximization functions were included at key steps along the cascade. While the drop off function allows health workers to rapidly assess how many patients were lost at each step, the maximization function details the additional people served if only one step improves to 100% capacity while others stay constant.

**Conclusions:**

Our experience suggests that adaptation of a cascade analysis tool for facility-level pMTCT services is feasible and appropriate as a starting point for discussions of where to implement improvement strategies. The resulting tool facilitates the engagement of frontline health workers and managers who fill out, interpret, apply the tool, and then follow up with quality improvement activities. Research on adoption, interpretation, and sustainability of this pMTCT cascade analysis tool by frontline health managers is needed.

**Trial Registration:**

ClinicalTrials.gov NCT02023658, December 9, 2013

## Background

Prevention of mother-to-child transmission (pMTCT) using antiretroviral prophylaxis is a proven, efficacious intervention, and can reduce vertical HIV transmission to less than 2% [1–3] In practice however, gaps along the pMTCT care cascade diminish pMTCT effectiveness in resource-limited settings.

The pMTCT cascade represents a complex system of sequential, interdependent steps that pregnant HIV-infected women pass through in order to receive appropriate care and treatment for themselves and their newborns. The sequential pMTCT ‘cascade’ typically includes antenatal care (ANC) attendance, HIV counseling and testing, prophylactic antiretroviral medicines (ARVs), safe delivery, safe infant feeding, infant follow-up including HIV testing, and family planning [4, 5]. The pMTCT cascade may also include women’s linkages into long-term HIV care and treatment services, including assessment for combination antiretroviral therapy (cART) and cART initiation.

Mozambique’s pMTCT cascade performance is similar to many resource-limited settings with a high HIV burden. HIV testing in ANC is high (estimated at 87% among ANC attendees), while maternal access to ARV prophylaxis and cART for eligible women and newborns remains low (51% and 42%, respectively) [6, 7]. Infant feeding practices, low post-partum use of modern family planning methods, weak linkages with HIV care, and sub-optimal integration with other effective ANC services further impede pMTCT effectiveness in Mozambique. As a result, pediatric HIV infection remains high in the country, with HIV transmission occurring in an estimated 28% of babies born to HIV-infected women [7]. The high rate of MTCT in Mozambique highlights the urgent need to improve the delivery of efficacious interventions to pregnant women and their infants.

Quality improvement (QI) has been highlighted as an approach to bridge the gap between evidence-based knowledge and its application to improve health outcomes [8, 9]. QI has been broadly applied in high resource health systems, and is increasingly being utilized in resource-limited settings, including maternal and child health services. QI has recently been applied to pMTCT [10], data quality [11, 12], continuous monitoring and performance improvement [12, 13], strengthening community/health facility linkages, and strengthening the coordination of non-governmental organizations (NGOs) [14]. However, typically QI for pMTCT has relied on time and resource intensive peer-to-peer exchange, training, and expert mentoring to successfully highlight delivery bottlenecks, identify workflow modifications to address these bottlenecks, and evaluate the impact of these innovations [12, 13, 15, 16]. Other studies have used quantitative analysis to assess the flow of patients through HIV services to highlight where to intervene for improvement efforts, though the complexity of the analysis procedures limits their use by clinic-level health workers [17, 18]. Simple strategies and tools to support heath authorities to independently conduct QI for pMTCT – including assessing where to intervene along the pMTCT cascade – are also needed to expand QI efforts and improve service effectiveness and program ownership. Specifically, a comprehensive systems view enables effective, evidence-based decision-making among managers and frontline health workers, including the identification of bottlenecks, and the definition and implementation of appropriate solutions to improve their system [19].

Use of routine health management information systems (HMIS) for decision-making has been difficult in resource-limited settings, including for pMTCT [18, 20, 21], partially due to lack of investment in HMIS quality [11]. Efforts to improve the quality of routine HMIS and stimulate its use by health system managers include periodic data audits [11, 18, 22, 23], the collection of fewer, streamlined indicators [24], and developing tools, such as the pMTCT cascade analysis tool (PCAT) tailored to the needs and competencies of health facility managers and frontline health workers to foster their active engagement with their data.

The objective of this paper is to introduce the process for developing a rapid, simple approach to support health facility managers to use routine HMIS data to assess facility performance across the pMTCT cascade as a first step in identifying where in the pMTCT cascade to intervene to maximize improvements in overall pMTCT delivery. In addition, this paper will address the selection of specific data content for the tool, which aims to increase health workers’ overall understanding of their health system flow, and the use of their routinely collected administrative data.

## Methods

### Scope and purpose

The overall objective of the pMTCT cascade analysis tool (PCAT) is to provide frontline MCH health managers at the health facility level with the means to rapidly, independently and quantitatively track patient flows through the pMTCT cascade, and readily identify priority areas for clinic-level improvement interventions.

### Setting

**Figure 1:**
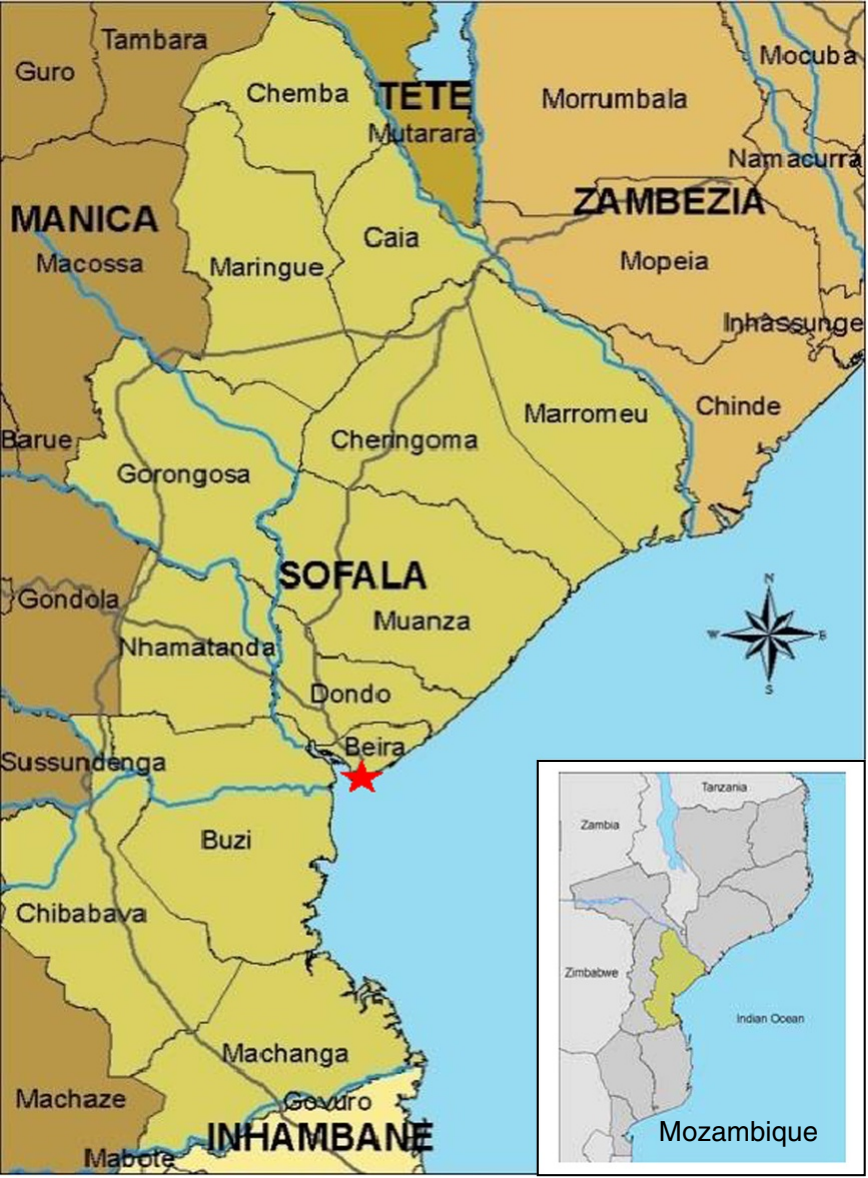
**Map of Sofala Province, Mozambique.**

The PCAT was developed, piloted, and refined over a 6-month period in Beira city, the capital of Sofala Province, central Mozambique (Figure 1). Beira is a large port city (population 470,000) [25], with a high HIV burden (estimated at 17.8%) [26]. In the Mozambique health system, maternal and child health (MCH) nurses are responsible for the management and delivery of pMTCT services across the pregnancy, birth and post-partum periods.

### Cascade tool development

Over a four-week period beginning in January, 2013, a group of 5 experienced MCH program managers and researchers met weekly to adapt an HIV care cascade tool for the pMTCT cascade [17]. This original tool included quantifying loss to follow-up across the multiple, linked steps in the HIV care cascade, including (1) HIV testing, (2) enrollment at an ART clinic for HIV-infected adults, (3) CD4 testing post enrollment, (4) ART initiation among eligible individuals, and (5) adhering to ART post ART initiation. The PCAT also quantifies the number of women that would complete the entire HIV care cascade if drop-off at each step were eliminated; this maximizing function allows health workers to quickly prioritize steps along the cascade to target for improvement.

In developing the PCAT, initial priorities included 1) introduction of the steps specific to the pMTCT cascade; 2) adaptation of the interface for data entry by frontline health managers using a broadly available program such as Excel®; 3) creation of versions in English, French and Portuguese; 4) ensuring the tool uses only data available through the routine HMIS; and 5) an output design that clearly indicates which individual improvement step would lead to the largest overall efficiency gains across the pMTCT cascade.

Process mapping techniques were used to chart PMTCT cascade steps in five facilities, including ANC attendance, HIV testing and counseling, provision of prophylactic ARVs, safe delivery, safe infant feeding, infant follow-up including HIV testing, and family planning, in order to obtain site-specific knowledge of service delivery [4, 27]. Women’s linkages into long-term HIV care and treatment services, including eligibility assessment for cART and cART initiation, were also considered as part of the pMTCT cascade. Study teams worked with staff from the ANC, maternity, postpartum, and at-risk child care settings over a number of days to draw maps of the flow of mother-infant pairs across these services. By working with facility staff to explicitly describe the sequential, linked processes of care delivery at their facility, key steps in the pMTCT cascade were highlighted [28].

After mapping the pMTCT cascade, study staff conducted discussions with health facility staff to refine the objectives of the PCAT, identify users and beneficiaries, and reach consensus on which steps of the pMTCT cascade should be included based on data availability, data quality, and their importance for achieving effective pMTCT service delivery.

Initial versions of the PCAT (designed in Excel®) were shared and discussed with 27 additional pMTCT managers and frontline nurses, including at the provincial (4 people), district (5 people from 3 districts), and facility levels (18 people from 10 health facilities). The tool was presented in a series of 1–2 hour meetings in which its understandability, usability, and appropriateness were discussed. Over the following six months the PCAT was redesigned based on continued stakeholder feedback, and pilot tested in five health facilities with pMTCT services before its introduction. The iterative development process is described in Table 1.

**Table 1:** **PCAT tool development timeline**

Activity	Month					
	1	2	3	4	5	6
Initial planning meetings-researchers/program managers	X					
Tool adaptation & development		X				
Feedback meetings with program managers & frontline health workers		X				
Tool revisions			X			
Feedback meetings with program managers & frontline health workers			X			
Tool revision				X		
Feedback with program managers & frontline health workers					X	
Tool introduction						X

## Results and discussion

**Figure 2:**
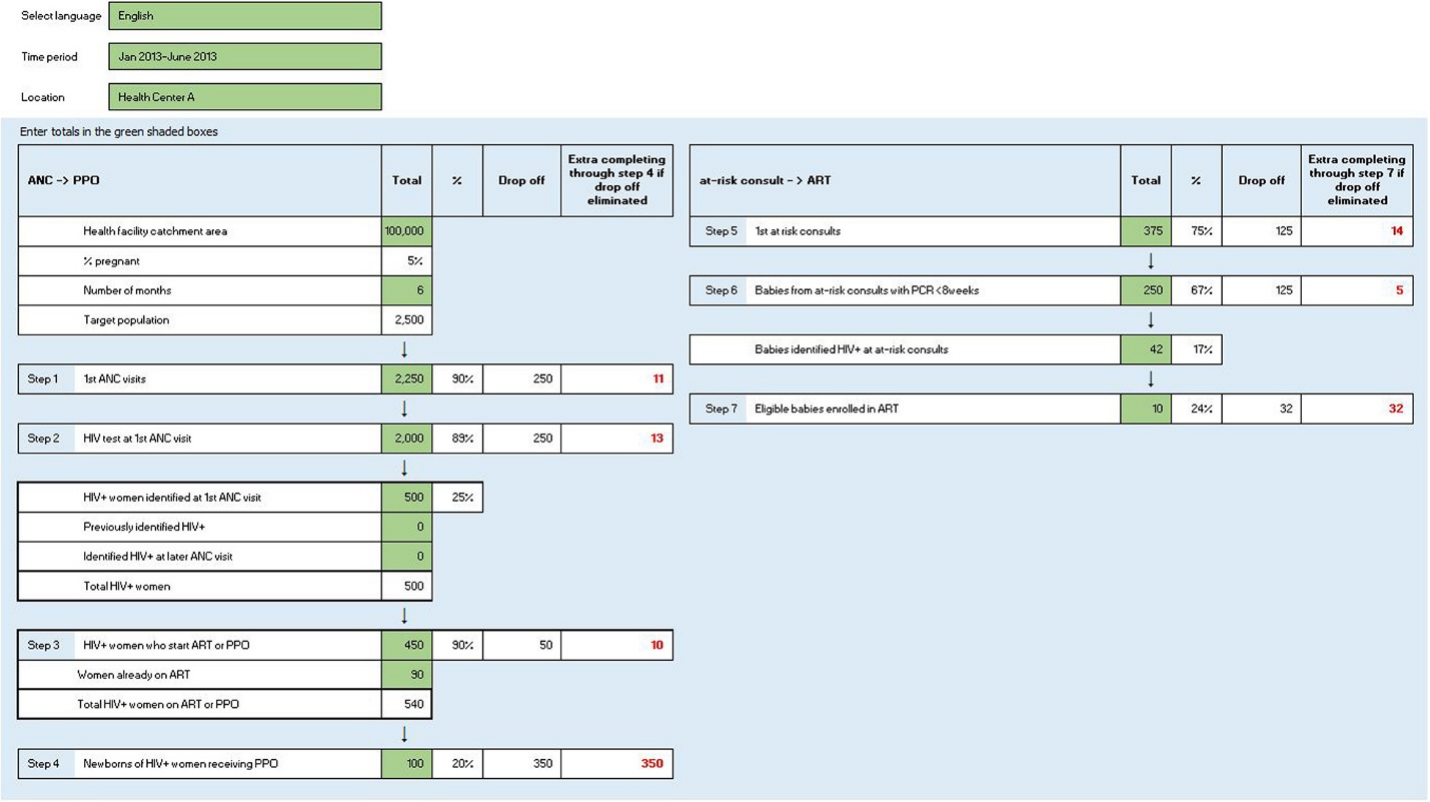
**PMTCT cascade analysis tool (screen shot).**

The first version of the PCAT was described by pMTCT managers as overly complex, requiring multiple data manipulations across multiple spreadsheets. Unlocked cells led to inadvertent formula tampering. CD4 testing was eliminated as a cascade step because the introduction of new pMTCT norms no longer required CD4 testing to determine cART or prophylactic ART eligibility [29]. Managers provided feedback that they preferred a tool which they could independently manipulate without technical guidance.The PCAT evolved in a number of notable ways (Figure 2). A total of seven cascade steps were included to cover two connected parts of the cascade (pregnancy through post-delivery, and delivery through infant follow-up). Prevalence calculations were included as sub-headings under relevant steps. Cells that did not require data inputs were locked and wording simplified. Font color, size and other design changes were made to improve PCAT user-friendliness.

Stepwise drop offs and maximization functions were included in the key steps along the cascade. Maximization functions were included for two different endpoints; for steps 1–4, step 4 (the number of newborns receiving ARV prophylaxis at the maternity) was the endpoint, while for steps 5–7, step 7 (the number of eligible babies who initiate ART) was the endpoint. While the drop off function allows the health worker to rapidly assess how many patients are lost at each step, the maximization function describes how many additional people will be served if only one step is improved to 100% capacity while others stay the same.

After the second feedback meeting, the PCAT was further simplified. The subsequent version was developed to streamline commands, add custom functions, and simplify the interface. Stakeholders universally preferred the revised PCAT visually and functionally, and found the tool to be easy-to-understand and useful. The final version of the tool for use and adaptation is located in Annex 1.A hypothetical example of the PCAT over a six-month period is presented in Figure 2, based on a facility catchment population of 100,000 and an estimated 5% prevalence of pregnancy. If the health facility reported 2,250 first ANC visits, then the tool will estimate that 90% of the target catchment population is served (a drop-off of 250 women), and that if this step were maximized to 100% (from 90%) then an additional 23 newborns of HIV infected mothers would receive prophylaxis at the maternity (step 4). The coverage, drop off, and maximization is quantified for each step along the cascade.

### PCAT content

The PCAT allows frontline health managers to rapidly quantify the drop off along the pMTCT cascade, and enables managers to quickly and easily identify which step has the largest impact on progression through the cascade (tool available at: http://www.healthallianceinternational.org/publications/presentations/).

#### Pregnancy through post-delivery

The first section of the PCAT begins at first ANC visit and extends to the administration of prophylactic ARVs for the newborn at the maternity. The initial four steps include (1) number/proportion of 1^st^ ANC visits (of those births expected); (2) number/proportion of HIV tests administered at the 1^st^ ANC visit; (3) number/proportion of women identified with HIV in the first ANC visit who initiate cART or prophylaxis (PPO); and the (4) number/proportion of newborns of HIV-infected mothers who receive prophylaxis at birth or shortly thereafter.

#### Post-delivery through ART initiation in children

The second cascade section begins post-delivery with the first at-risk newborn consult through the administration of cART for eligible HIV-infected infants. The three steps of this section of the cascade include (5) number/proportion of first at-risk child visits among HIV-exposed newborns; (6) number/proportion of enrolled children with PCR tests completed prior to 8 weeks of life; and (7) number/proportion of eligible children initiating cART.

##### Step 1: Number of 1st ANC visits

Data on the number of first ANC visits from registries or facility reports is divided by the target population to estimate the proportion of pregnant women accessing ANC. The drop off (or added number gained if this step were to be maximized) is calculated by subtracting the number of first ANC visits from the estimated target population.

##### Step 2: Number of HIV tests at 1st ANC visit

Data on the number of women receiving HIV testing in first ANC is entered to estimate the proportion of pregnant women tested for HIV, with the number to be gained if this step were improved calculated by subtracting the number receiving an HIV test from the number of first ANC visits. HIV testing data are located in ANC registries and in facility reports. HIV testing in subsequent ANC visits are not included in this calculation, as its objective is to model actual performance compared with a fully functioning system. Although women are often tested for HIV later in ANC, they would ideally be tested at first visit to have time for comprehensive pMTCT services prior to delivery. Therefore, the maximizing function for this step assesses the number of additional women who would be tested in first ANC visit should this step be improved.

After entering the number of women tested for HIV, the tool requires entering the number of HIV-infected women identified, and automatically calculates HIV prevalence. Additional fields allow for entering the number of women identified with HIV in prior pregnancies and subsequent ANC visits to provide an accurate denominator for further steps in the pMTCT cascade.

##### Step 3: HIV + women who start PPO or cART

The total number of pregnant women initiating cART or PPO over the relevant time period is entered, and the proportion of HIV + women receiving an effective PPO or cART during ANC is automatically calculated. If this step were improved, the number of additional women receiving PPO or cART is calculated by subtracting the number receiving PPO or cART from the number of HIV-infected women attending ANC. Required data are available in ANC registries and monthly facility reports.

##### Step 4: Newborns of HIV + women receiving PPO

The number of newborns of HIV-infected mothers who receive PPO at the maternity directly after birth is entered and the proportion of newborns of HIV + mothers receiving PPO at the maternity is automatically calculated. The number of additional HIV-exposed newborns who would receive PPO if this step were maximized is calculated by subtracting the number receiving PPO from the total number of HIV-infected women in antenatal care. Required data are available in maternity registries and monthly facility reports.

##### Step 5: 1st at-risk child visits

The number of HIV-exposed children who enroll at at-risk child care is entered to estimate the proportion of children born to women identified with HIV in pregnancy who enter HIV care. The number of additional HIV-exposed newborns who would enroll in HIV care if this step were improved is estimated by subtracting successfully enrolled children from the overall number of children born to HIV-infected women identified in ANC. These data are available in the at-risk child consult registry which informs the monthly facility level report and is subsequently entered into the electronic HMIS.

##### Step 6: Babies in at-risk care who receive a PCR <8 weeks

The number of children enrolled in at-risk care who receive an HIV diagnosis via polymerase chain reaction (PCR) by 8 weeks of life is entered to calculate the proportion of enrollees with an early HIV diagnosis, and estimate drop off in children who receive a HIV diagnosis according to recommendations if this step were improved. Data on HIV diagnosis by PCR is captured via clinic registries and monthly health facility level reports. Subsequently, the number of HIV-infected newborns identified through PCR testing within 8 weeks of life is entered to calculate the proportion of HIV-infection in tested infants, and provide a denominator for the last cascade step.

##### Step 7: Eligible babies enrolled in cART

The number of children initiating cART is used to estimate the proportion of eligible children initiating cART, and the added number who would initiate cART if this step were improved. These data are sourced from clinic registries and monthly health facility reports.

Study procedures were approved by the Ethics Committee of the Mozambique Ministry of Health, and was determined to be non-research by the University of Washington Institutional Review Board.

## Conclusion

This paper describes the development process and content of a systems analysis tool for pMTCT services (PCAT). The PCAT is intended to be a decision-making support tool for front line health workers and district managers by providing a comprehensive understanding of their systems’ performance, covering ANC, delivery, and post-partum care for mothers and their newborn children. After iterative testing, the PCAT was streamlined to capture key steps of the pMTCT cascade, be user-friendly for stakeholders in resource-constrained environments, and be used as adjunctive support for quality improvement in pMTCT.

A primary strength of the PCAT is that it was designed for use in settings with limited human and financial resources and high HIV burden. By relying on routinely-collected data (or data available through clinic registries), the PCAT can be readily populated to provide a rapid guide on where to intervene in the pMTCT cascade to maximize the number of women and children receiving successful pMTCT. The PCAT is open-source, available in English, French and Portuguese, and is highly flexible to meet the needs and context where it is intended to be applied. The PCAT can use data collected over any duration (such as one month, six months or one year), or aggregate the level of the health system (facility, district or province) depending on the needs of the user. The PCAT is meant to complement ongoing data quality audits as well as facility-based quality improvement efforts, by encouraging health workers to incorporate improved administrative data into their decision-making.

### Lessons learned through PCAT development

The PCAT development process and resulting tool provides a number of lessons learned that influence its use for quality improvement efforts.

#### Maximizing function

Numerous papers have documented the use of cascade drop off, specifically identifying the number of eligible patients not receiving services by cascade step. However the addition of the maximizing function provides health facility managers with information on how many additional people will be served if only one step is improved to 100% capacity while others stay the same. This information can be extremely helpful in identifying where in the pMTCT cascade interventions to intervene. Although not a panacea, the PCAT can be a powerful tool for health facility managers who are most able to make a difference in their workplace service flow.

#### Data quality

Because it will influence service delivery modifications, the PCAT requires data of acceptable quality, which can be a challenge in resource-constrained health systems facing high HIV burden. For example, over- or under-estimates of utilization may result from inaccurate catchment population estimates. By engaging frontline health workers and managers in PCAT implementation, the process is designed to focus attention first on areas of potential or actual poor data quality (such as unrealistic numbers), with the intention of improving data quality. By acknowledging the poor quality of the data they can go back to the registries to identify the source of the discrepancy.

#### Making choices

To encourage its use, the tool was pared down to a minimum number of steps within the PMTCT cascade. Because the PCAT is designed to guide interventions in pMTCT within ANC, maternity, and at-risk child visits, these phases were included in the PCAT. Critical areas such as family planning, linkages to long-term ART for mom and baby, and community linkages were not included. These are areas of great importance, which merit further study and adaptation but were not within the scope of this paper.

In addition, CD4 testing was not included in this cascade for two reasons. With the advent of Option B+, which places all HIV-infected mothers on ART regardless of CD4 levels, this measure is no longer a critical entry step in the pMTCT cascade. In addition, a transition of partner support in the study area resulted in extremely limited data availability for this measure. As a result, over half of the sites reported no available data, which made beta testing untenable.

#### Shopping around

In large, urban areas with multiple health facilities, we found that it is common for pregnant and post-partum women to seek services at multiple facilities depending on factors such as proximity, desire for anonymity, perceived service quality, and availability of specialized services. Thus, a woman may attend one facility for ANC, another for birth and still another for at-risk care for newborns. As a result, in urban areas the PCAT may under or over-estimate service utilization across the pMTCT cascade at the facility level. However, discussion based on the PCAT can lead to an appreciation of these dynamics by facility staff, and based on iterative application of the PCAT with quality improvement activities, the PCAT data may become more accurate as retention improves at individual facilities over time.

#### Shifting pMTCT strategies and forms

The PCAT development process coincided with the introduction of the Option B + approach for pMTCT in Mozambique, which changes the existing strategy to initiate lifelong cART for pregnant women after HIV diagnosis [30]. This change has simplified the PCAT by eliminating CD4 as an entry barrier for cART or PPO. Furthermore, new forms were introduced during PCAT development, including MCH ‘passports’ that include longitudinal patient data for mothers and children, new clinic registries and monthly reporting formats. In some instances these modifications were designed to address needs for patient-level care indicators and donor requirements rather than systems strengthening efforts. An example of this is the selection of the number of CD4 tests under 350 cells/mm^3^ rather than the total number of CD4 tests carried out, which is an essential indicator for assessing pMTCT system functioning prior to the introduction of Option B+. The PCAT is sufficiently flexible to address changes in clinical protocols and changes in reporting format.

Our experience suggests that it is feasible and appropriate to adapt this cascade analysis tool for pMTCT services at the health facility level as a starting point for discussions of where to implement improvement strategies. The resulting PCAT does not stand alone, but requires engagement with frontline health workers and managers to fill out, interpret and apply the tool, and then follow up with QI activities. In addition, data quality audit activities support the strengthening of the administrative source data. Further research on how to encourage adoption, interpretation, and sustainability of the PCAT by frontline health workers and managers is needed. However, the PCAT is available for application and further refinement, and provides a platform to improve the use of data for decision-making as an interim step towards improving service delivery and patient health outcomes.

## Abbreviations

ANC: Antenatal Care
ART: Antiretroviral Therapy
ARV: Anteretrovirals
cART: Combined Antiretrovirals
CD4: Cluster of Differentiation 4
HIV: Human Immunodeficiency Virus
HMIS: Health Management Information System
MCH: Maternal and Child Health
NGO: Non-Governmental Organization
PCAT: PMTCT Cascade Analysis Tool
PCR: Polymerase Chain Reaction
pMTCT: Prevention of Mother-to-Child Transmission
PPO: Prophylaxis (to prevent HIV transmission)
QI: Quality Improvement.
